# Personality traits and anxiety in patients with temporomandibular disorders

**DOI:** 10.1186/s40359-022-00795-8

**Published:** 2022-04-04

**Authors:** Atefeh Hekmati, Nazanin Mortazavi, Rahman Berdi Ozouni-Davaji, Mohammadali Vakili

**Affiliations:** 1 Dental Research Center, Golestan University of Medical Sciences, Gorgān, Iran; 2 Department of Oral and Maxillofacial Medicine, School of Dentistry, Golestan University of Medical Sciences, PO Box 4916953363, Gorgān, Iran; 3 Health Management and Social Development Research Center, Golestan University of Medical Sciences, Gorgān, Iran; 4 Health Management and Social Development Research Center, Department of Biostatistics and Epidemiology, Faculty of Health, Golestan University of Medical Sciences, Gorgan, Iran

**Keywords:** Anxiety, Personality disorders, Temporomandibular disorders

## Abstract

**Background:**

Temporomandibular disorders (TMD) have long been suggested to result from psychological factors. Recent studies, however, tend to consider TMD a chronic psychosomatic illness. The present study was designed to explore the association between TMD and personality profile. The Minnesota Multiphasic Personality Inventory-2-Reconstructed form (MMPI-2-RF) was used to evaluate the association for the first time.

**Methods:**

A total of 258 subjects participated in this case–control study. TMD cases as detected by the Helkimo index were questioned regarding their personality characteristics and anxiety levels using MMPI-2-RF and Spielberger state and trait anxiety inventory.

**Results:**

Patients with TMD scored higher on all personality characteristics except for Aberrant Experiences. The psychological profile of TMD showed no significant difference between theoretical and experimental Ideas of Persecution means. Patients with TMD reported significantly higher mean levels of state and trait anxiety than controls. The most frequently found anxiety levels in TMD cases have been mild state and trait anxiety (77.5% versus 74.4%).

**Conclusion:**

Personality characteristic scores were considerably higher in TMD patients. TMD cases detected by Helkimo index manifest both trait and state anxiety as common findings.

## Background

Chronic pain is a complex phenomenon, involving both psychological and physical aspects. Chronic pain has been studied for a long time using various behavioral and personality inventories [1]. In particular, chronic pain is conceptualized as multidimensional with sensory, cognitive, and social influences. A psychometric instrument called Minnesota Multiphasic Personality Inventory (MMPI) was designed to differentiate between the psychological and physical causes of chronic pain by identifying the personality traits common to these individuals [2]. Minnesota Multiphasic Personality Inventory-2-Reconstructed form (MMPI-2-RF) is the most recent version of MMPI, which was revised and concised as compared to MMPI-2 (338 vs. 567 items). MMPI-2RF was developed to assess the contemporary models of personality and psychopathology. Practitioners find this tool useful in detecting individuals who pretend to have physical health problems [3].

The assumption that psychological factors can contribute to temporomandibular joint disorder (TMD) has been developed during the 1950s [4]. Since then, the influence of emotional traits on TMDs has received much attention [5]. Psychological functioning has been associated with the duration of TMD pain, as with other types of pain [6]. A comparison of TMD and control subjects by Ferrando et al. revealed different psychological characteristics [7]. The TMD cases are typically more prone to stress despite being no more anxious than controls [8]. Even so, many TMD patients are not aware of their emotional states [9]. There is however a need to pay attention to the multiple aspects of TMD in order to ensure their quality of life and general health [10].

The American researchers recently found that somatic symptoms are strongly related to TMD onset [11]. In addition, psychological factors have a more significant impact on TMD pain that is muscular in origin [12, 13]. A subjective pain measure for TMD patients developed by Martti Helkimo in 1974 is called an anamnestic index [14, 15].

The use of various inventories to measure personality traits in these patients, including earlier forms of the MMPI, has been widely discussed. Orofacial pain has been discussed in terms of older versions of the inventory, that is, the MMPI and MMPI-2 [5]. In this study, we apply MMPI-2-RF to assess personality traits in TMD cases for the first time. Additionally, Spielberger state and trait anxiety inventory (STAI) was used in the present study to assess patients' anxiety.

## Methods

A case–control study was conducted to assess the anxiety and personality traits of random outpatients visiting the Golestan University of Medical Sciences (GOUMS). The inclusion criteria were as follows:

1. Conscious participation in the study.
2. Adults aged 18 years old or older (80 years at most), according to MMPI-2-RF manual.
3. No systemic disease affecting the lower jaw (e.g. RA, Scleroderma, Sarcoidosis, Psoriasis, Behcet’s disease).
4. No previous trauma to the mandible.
5. No previous orthodontic treatment.
6. No unilateral or bilateral loss of four posterior teeth.

No exclusion criteria apart from the inclusion criteria was defined. Cases of TMD were detected with Helkimo index (anamnestic component [AI] and clinical dysfunction component [Di]). Controls were also selected from GOUMS visitors who did not have TMD and were matched on the basis of their gender and age (5 years). A total of 129 individuals participated in each group. All methods were performed in accordance with the relevant guidelines and regulations. Several variables were used to analyze this study, including the following: sex, age, educational level, personality traits (characteristics) based on the MMPI-2-RF, anxiety based on the STAI, and detection of TMD, based on the Helkimo index.

A Helkimo index identifies TMD, maximal mouth opening, jaw deviation, TMJ function, and TMJ/muscle pain. The questionnaire was designed to enable calculation of the Helkimo anamnestic index (Ai) based on subjective feelings and positive or negative answers of subjects regarding the state of their masticatory apparatus. Another component of the questionnaire, the Helkimo clinical dysfunction index (Di) offers an objective functional evaluation of structural and functional disorders of the orofacial complex. The severity of TMD is determined by the sum of the measurements of approximately 25 points. Each individual had a total dysfunction score ranging from 0 to 25 points. A higher score indicates a more acute/serious disorder. Depending on the values obtained, the patients were classified as follows: Di0 – no dysfunction; DiI – mild dysfunction (1–4 points); DiII – moderate dysfunction (5–9 points); DiIII – severe dysfunction (9–25 points) [14, 15].

MMPI-2-RF is a revised instrument comprising 338 items designed to represent the clinically significant items in the MMPI-2. There are six sets of scales in the test, including the Validity, Higher-Order (H-O), Restructured Clinical (RC), Specific Problems, Interest, and Personality Psychopathology Five scales. The H-O scales consist of Emotional/Internalizing Dysfunction (EID), Thought Dysfunction (THD), and Behavioral/Externalizing Dysfunction (BXD). Scales of RC are composed of Demoralization (RCd), Somatic Complaints (RC1), Low Positive Emotions (RC2), Cynicism (RC3), Antisocial Behavior (RC4), Ideas of Persecution (RC6), Dysfunctional Negative Emotions (RC7), Aberrant Experiences (RC8), and Hypomanic Activation (RC9) [3]. As part of the current study, we focused on the H-O scales as well as the RC scales that represent broad psychopathological and personality dimensions.

The STAI consists of two sets of twenty items, which provide scores that indicate both the level of anxiety the subject has at present (state) and the extent to which the person is prone to experience anxiety (trait). State anxiety items include: “I am tense; I am worried” and “I feel calm; I feel secure.” Trait anxiety items include: “I worry too much over something that really doesn’t matter” and “I am content; I am a steady person.” All items are rated on a 4-point scale (e.g., from “Almost Never” to “Almost Always”). All items are rated on a 4-point scale (e.g., from “Almost Never” to “Almost Always”). The levels of anxiety are interpreted as normal or no anxiety; 10–18, mild to moderate anxiety; 19–29, moderate to severe anxiety; and 30–63, severe anxiety [8, 16]. The sample size required for the current study was determined based on a study conducted by Meldolesi et al. [9]. It was determined that 129 subjects for each group, resulting in a total of 258 subjects, were required to achieve a confidence interval (CI) level of 0.95, 80 percent power for analysis, and minimal error.

### Statistical analyses

SPSS version 18 was used for describing the means, standard deviation, ranking means, frequency, and percentage. Participants were assessed using MMPI-2-RF and STAI. An independent t-test or non-parametric Mann–Whitney test was used to compare anxiety levels and personal characteristics. A Chi-square test was used to test qualitative variables such as ranking means for anxiety levels. Correlations between anxiety, personality characteristics and TMD clinical dysfunction were examined by Spearman correlation coefficient. All analyses were considered significant at the level of 0.05.

## Results

A total of 258 individuals participated in the study. Among the subjects were 130 men (50.38%) and 128 women (49.62%). The TMD group consisted of 65 men (50.38%) and 64 women (49.61%). In the healthy control group, the gender distribution was the same. The age range was 21 to 25 years (28.98 ± 7.01). The case group's age was 28.98 ± 6.93 years compared to 28.98 ± 7.25 years in the control group. A non-parametric Mann–Whitney test showed no significant difference between TMD and control group. In terms of education level, 63.8% of control and 71.3% of patient groups had a bachelor's degree or higher. According to the chi-square test, the difference in educational level—including those with less than a bachelor's degree—was non-significant.

The proportions of TMD patients with relatively mild, relatively severe, and mild traits of anxiety were 77.5%, 19.4%, and 3.1%, respectively. Similarly, 74.4%, 22.5%, and 3.1% of the TMD cases also had state anxiety that was relatively mild, relatively severe, and mild, respectively. TMD patients showed significantly higher means of both trait and state anxiety compared with controls (P-value < 0.0001). Spearman correlation coefficients indicated a significant linear relationship between dysfunction components of TMD and state anxiety (correlation coefficient = 0.32, P-value < 0.0001). The dysfunction components of TMD and trait anxiety also exhibited a significant linear relationship (correlation coefficient = 0.35, P-value < 0.0001).

In regards to the H-O scales, there was a significant difference between the experimental and theoretical means in TMD case (P-value < 0.0001; one-sample t-test). Experimental means were lower than theoretical means in the present study. In other words, H-O scales were below the expected level or average. Other personality characteristics, with the exception of Persecution Ideas, had experimental means significantly less than theoretical means, as shown in Table 1. Therefore, Ideas of Persecution were about average, while other characteristics were below average.

**Table 1:** Evaluation of personality characteristic Scores in TMD cases

Personality characteristic scores	Theoretical mean	Experimental mean	P-value
Demoralization	65	59.18	< 0.0001
Somatic complaints	65	58.89	< 0.0001
Low positive emotions	65	58.79	< 0.0001
Cynicism	65	59.43	< 0.0001
Ideas of persecution	65	63.15	0.146
Dysfunctional negative emotions	65	59.12	< 0.0001
Aberrant experiences	65	58.49	< 0.0001
Hypomanic activation	65	51.03	< 0.0001
Antisocial behavior	65	49.02	< 0.0001

Personality features such as EID, BXD, Demoralization, Somatic Complaints, Low Positive Emotions, Antisocial Behavior, Ideas of Persecution, and Dysfunctional Negative Emotions, were more pronounced among TMD groups than those of controls (Tables 2 and 3). Spearman correlations revealed direct and significant relationships between TMD clinical dysfunction and Demoralization, Somatic Complaints, Low Positive Emotions, Cynicism, Antisocial Behavior, Ideas of Persecution, and Dysfunctional Negative Emotions with correlation coefficient of 0.32, 0.34, 0.24, 0.18, 0.28, 0.28 and 0.16, respectively (P-value = 0.01). There was no significant linear relationship between TMD clinical dysfunction and Aberrant Experiences, but TMD clinical dysfunction and Hypomanic Activation were inversely related (correlation coefficient = − 0.19; P-value = 0.003).

**Table 2:** Comparison of personality characteristic scores in TMD cases/controls

Studied groups							Statistics
Personality characteristic scores	Case			Control			Mann–Whitney U P-value
	Mean	Std. Dev	Ranking Mean	Mean	Std. Dev	Ranking Mean	
Demoralization	59.18	6.63	151.80	54.36	6.01	107.20	5443.500 < 0.0001
Somatic Complaints	58.89	8.58	154.21	52.99	9.05	104.79	5132.500 < 0.0001
Low Positive Emotions	58.79	9.66	144.49	54.58	8.13	114.51	6387.000 0.001
Cynicism	59.43	7.60	137.92	58.23	5.50	121.08	7234.000 0.050
Ideas of Persecution	63.15	13.25	143.78	59.48	4.37	115.22	6478.500 0.002
Dysfunctional Negative Emotions	59.12	8.22	138.74	56.65	6.54	120.26	7129.000 0.045
Aberrant Experiences	58.49	10.64	124.61	59.15	7.04	134.39	7690.000 0.287
Hypomanic Activation	51.03	7.83	111.43	54.17	5.10	147.57	5989.000 < 0.0001
Antisocial Behavior	49.02	7.16	146.02	45.91	4.73	112.98	6189.500 < 0.0001

**Table 3:** Comparison of higher-order scales in TMD cases/controls

Studied groups							Statistics
Score	Case			Control			Mann–Whitney U P-value
	Mean	SD	Ranking Mean	Mean	SD	Ranking Mean	
Emotional/internalizing dysfunction (EID)	56.86	8.08	157.99	50.48	5.40	101.01	4645.500 < 0.0001
Thought dysfunction (THD)	51.31	7.30	113.72	54.04	4.73	145.28	6285.000 0.001
Behavioral/externalizing dysfunction (BXD)	59.51	12.66	135.27	57.15	5.70	123.73	7576.000 0.204

According to the Helkimo index, anamnestic evaluations of TMD subjects revealed that 58.91%, 24.8%, and 41.86% of them experienced joint sound, pain, and fatigue (Table 4). Within the Clinical dysfunction component, 58.13% had limited mouth opening, 22.48% had locked mandibles, and 24.80% had jaw deviation (Table 4).

**Table 4:** Prevalence of signs and symptoms among TMD cases

INDEX	Men	Women	Total
	Number (%)	Number (%)	Number (%)
Anamnestic component			
TMJ sound	38 (50%)	38 (50%)	76 (58.91%)
Facial and jaw pain	16 (50%)	16 (50%)	32 (24.80%)
Jaw and masticatory muscle fatigue	27 (50%)	27 (50%)	54 (41.86%)
Clinical dysfunction			
Limited mouth opening	37 (49%)	38 (51%)	75 (58.13%)
Locked mandible	12 (41.37%)	17 (58.62%)	29 (22.48%)
Jaw deviation	19 (59.37%)	13 (40.63%)	32 (24.80%)

There was mild, moderate, and severe dysfunction in 23.64%, 20.54%, and 5.81% of the TMD subjects, respectively. Among the subjects, the majority (61.24%) were symptom-free, followed by 18.21% with mild symptoms, and 22.09% with severe symptoms (Table 5).

**Table 5:** Evaluation of TMD severity by Helkimo index

INDEX	Men	Woman	Total
	Number (%)	Number (%)	Number (%)
Anamnestic index			
Ai 0 (free of symptoms)	77 (50%)	77 (50%)	154 (61.24%)
Ai I (mild symptoms)	24 (51.07%)	23 (48.93%)	47 (18.21%)
Ai II (severe symptoms)	29 (50.88%)	28 (49.12%)	57 (22.09%)
Dysfunction component			
Di 0 (no dysfunction)	65 (50.39%)	64 (49.61%)	129 (50%)
Di I (mild dysfunction)	32 (46.52%)	29 (47.54%)	61 (23.64%)
Di II (moderate dysfunction)	27 (50.97%)	26 (49.05%)	53 (20.54%)
Di III (severe dysfunction)	6 (40%)	9 (60%)	15 (5.81%)

## Discussion

TMJ literature indicates increased levels of stress and anxiety in TMD cases. According to the STAI, TMD patients showed a markedly greater anxiety level compared with control participants in the present study. Our study was consistent with a study by Vojdani and her colleagues that demonstrated higher levels of state and trait anxiety in TMD patients than in healthy controls [16].

As a result of such components as personality characteristics, our study also reported significantly higher scores in TMD patients than in controls. Tables 2 and 3 listed H-O and RC personality characteristics that were more prevalent in the TMD group than in the control group. These are EID and BXD, Demoralization, Somatic Complaints, Low Positive Emotions, Antisocial Behavior, Ideas of Persecution, and Dysfunctional Negative Emotions. The results may not be compared to previous research, as MMPI-2-RF has not been used for TMD. The STAI and MMPI-2-RF findings of the present study can be understood in light of Auerbach’s comments that TMD cases are more likely to be exposed to stressful experiences in their lives [13].

With respect to personal characteristics such as BXD, Cynicism, and Aberrant Experiences in TMD and control groups, there were no significant differences between ranking means. In line with this, McNeil et al. found no difference between TMD and the control group in terms of those characteristics [17]. In our study, however, we found that the ranking means for Hypomanic Activation and THD were higher for controls than for TMD patients (Tables 2 and 3).

TMD patients and dysfunctional patients with higher levels of emotional problems show significant improvement when offered treatments such as stress management/biofeedback and intraoral appliances. This clearly shows that psychological factors have a significant effect on TMD [18, 19]. Similarly, Tversky and Reade provided supportive psychotherapy to TMD patients rather than occlusal splints and anti-depressants [20]. According to Blackburn et al., cognitive therapy was superior to drug treatment [21].

Additionally, our study did not differentiate between sample means of psychological profiles, such as anxiety and personality characteristics, according to the level of education. We may announce that education levels did not affect anxiety and personality characteristics. In a study by Adriani and colleagues, there was no difference between graduate students seeking a master's or a doctorate degree regarding anxiety levels or emotional stress; our results appear to be comparable [22]. Despite this, graduates from community colleges were more likely to experience anxiety than graduates from universities [23]. TMD patients may be caused to engage in pain-evoking behaviors as a result of adjunctive behaviors, such as uncertainty in their illness [24, 25].

## Limitation

Findings of the current study should be viewed in the context of limitations due to the length of the MMPI-2-RF questionnaire, which may have led participants to answer questions unrealistically or choosing answers at random.

Furthermore, the main limitation of the Helkimo index as a TMD assessment tool is that it does not differentiate between joint and muscle symptoms. Research diagnostic criteria for temporomandibular disorders (RDC/TMDs) and its modified version known as the Diagnostic Criteria for Temporomandibular Disorders (DC/TMD) include a valid and reliable screening questionnaire for diagnosing most common pain-related TMDs. It is recommended that DC/TMD be used in future studies.

## Conclusion

Based on the results of this study, there are significant differences between the means of State/Trait anxiety in TMD patients and controls. Trait anxiety and state anxiety are common findings in TMD cases detected by the Helkimo index. TMD patients' personality characteristic scores tend to be significantly higher than those of controls.

## Data Availability

The datasets generated and/or analyzed during the current study are not publicly available due to confidentiality of information but are available from the corresponding author on reasonable request.

## Abbreviations

TMD: Temporomandibular disorders
TMJ: Temporomandibular joint
MMPI-2-RF: Minnesota Multiphasic Personality Inventory-2-Reconstructed form
MMPI: Minnesota Multiphasic Personality Inventory
STAI: Spielberger State-Trait Anxiety Inventory
H-O: Higher-Order
EID: Emotional/Internalizing Dysfunction
THD: Thought Dysfunction
BXD: Behavioral/Externalizing Dysfunction
RC: Restructured Clinical
